# Vitamin D_3_ Protects Mice from Diquat-Induced Oxidative Stress through the NF-*κ*B/Nrf2/HO-1 Signaling Pathway

**DOI:** 10.1155/2021/6776956

**Published:** 2021-11-16

**Authors:** Haiwen Zhang, Youming Liu, Xin Fang, Lihong Gu, Caiwei Luo, Lu Chen, Qian Wang

**Affiliations:** ^1^College of Animal Science and Technology, Hainan University, Haikou, Hainan 570228, China; ^2^Hainan Academy of Agricultural Sciences, Haikou, Hainan 570228, China

## Abstract

Vitamin D_3_, as an indispensable and fat-soluble micronutrient, plays an important role in the health of humans and animals. At present, studies are focusing on the calcium absorption and immunoregulation function of vitamin D_3_; this study was aimed at exploring the antioxidative stress ability of vitamin D_3_ on diquat-induced intestinal dysfunction of ICR mice and the underlying mechanism. The results showed that oral gavage of vitamin D_3_ daily significantly improved the body weight gain and immune organ index and significantly reverted the abnormal changes of ALT, AST, SOD, GSH-Px, T-AOC, and MDA in the serum and jejunum induced by diquat. The addition of vitamin D_3_ also significantly reduced the concentration of DAO, D-LA, and certain proinflammatory cytokines in serum. Moreover, vitamin D_3_ improved the pathological morphology of the duodenum, jejunum, colon, liver, and kidney tissues, and it also largely attenuated the degree of inflammatory infiltration of macrophages and cell apoptotic index of jejunal epithelial tissue induced by diquat. The results demonstrated that vitamin D_3_ significantly recovered the intestinal barrier injury by enhancing the expression of mucins and tight junction proteins in the jejunum. In addition, the results indicated that vitamin D_3_ could significantly reduce the phosphorylation level of NF-*κ*B (p65) and enhance the expression of Nrf2 and HO-1 in the jejunum compared with the diquat-induced group. This study suggested that oral administration of vitamin D_3_ can protect mice against oxidative damage by inhibiting the phosphorylation level of NF-*κ*B (p65) and activating Nrf2-related signaling pathways.

## 1. Introduction

The active form of vitamin D_3_ (VitD_3_) is 1*α*,25-(OH)_2_-D_3_, which forms 25-hydroxyvitamin D_3_ under the catalysis of 25-hydroxylase in the liver and further forms 1*α*,25-(OH)_2_-D_3_ through the action of 1-*α* hydroxylase in the kidney [1]. VitD_3_ is a fat-soluble vitamin type that plays an important role in many physiological processes related to human health; the lack of VitD_3_ would inhibit the absorption of calcium and phosphorus in the small intestine and cause the reduction of bone mineral density, which increases the risk of fracture [2]. Apart from the nutritional effects, the immunoregulation function of VitD_3_ has also been widely reported. VitD_3_ has a high affinity for the nuclear vitamin D receptor (VDR), which could initiate the operation of vitamin D response elements and upregulate the transcriptional activity. Through the VDR-related pathway, VitD_3_ could be involved in regulating the innate and adaptive immune systems efficiently [3]. The confirmed evidence had demonstrated that VitD_3_ could effectively enhance the expression level of human LL-37 antimicrobial peptide in several cell types, which contributes to the anti-infective and antibacterial functions [4, 5]. VDR- and VitD_3_-related metabolic enzymes are broadly expressed among macrophagocytes, dendritic cells (DC), and activated lymphocytes, which further verifies the immunomodulatory effects of VitD_3_ [6]. VitD_3_ could reduce the secretion of M1-type proinflammatory cytokines and promote the polarization type from M1 to M2 [7]. The addition of VitD_3_ to mice with ulcerative colitis could ameliorate the inflammatory status by reducing the infiltration of macrophages [8]. It is also reported that VitD_3_ could downregulate the expression of MHC II, costimulatory molecules, and IL-12 to inhibit the differentiation and maturation of DC, finally blocking the activation of T lymphocytes [9]. As to the effects of VitD_3_ on lymphocytes, it was reported that VitD_3_ could suppress the proliferation of Th1 lymphocytes and reduce the secretion of proinflammatory cytokines such as IL-2, IL-6, and IL-17, while it could enhance the activity of Th2 lymphocytes and promote the expression of IL-4 and IL-10 [10]. Pretreatment of VitD_3_ on B lymphocytes could decrease the activation degree of CD40L and further reduce the production of T lymphocyte-originated proinflammatory cytokines [11].

The occurrence of oxidative stress (OS) in the body indicates the overproduction of reactive oxygen species (ROS) and will cause many diseases [12]. For instance, oxidative stress could destroy the structure of the intestine and lead to the dysfunction of the intestinal barrier. As a consequence, the inflammatory status will aggravate both the intestine and the whole circulatory system. The ROS leaked from the intestine may transfer to other parenchymal organs such as the liver, kidney, and spleen through blood, which could attack these organs and result in dysfunction [13]. Diquat (DQ), as an oxidant, is an ideal inducer to establish animal models of oxidative stress and has been widely used in different animals to duplicate the oxidative damage models successfully [14–16]. DQ could utilize molecular oxygen to produce a large amount of superoxide anion free radicals and attack the mitochondria to generate excess ROS [17], which could in turn cause oxidative damage and inflammation of the body.

The nutritional and anti-infective effects of VitD_3_ have been reported; however, there are few studies on the antioxidant stress function and the possible mechanism of VitD_3_. Therefore, this study was conducted to evaluate the protective effect of vitamin D_3_ on diquat-induced oxidative damage in the ICR mouse model; the NF-*κ*B/Nrf2/HO-1 signaling pathway was also investigated to explore the underlying mechanism of vitamin D_3_ exerting antioxidant effects.

## 2. Materials and Methods

### 2.1. Reagents

The active form of VitD_3_ (1*α*,25-(OH)_2_-D_3_) (Catalog number V8070) was purchased from Solarbio (Beijing, China) with a purity ≥ 99%. Diquat (Catalog number D101258) was purchased from Aladdin (Shanghai, China). The kits to determine the activity or concentration of aspartate aminotransferase (AST) (Catalog number MK5778A), alanine aminotransferase (ALT) (Catalog number MK5727A), superoxide dismutase (SOD) (Catalog number MK3125A), glutathione peroxidase (GSH-Px) (Catalog number MK5870A), total antioxidant capacity (T-AOC) (Catalog number MK4580A), and malondialdehyde (MDA) (Catalog number MK5892A) in the serum and jejunum were purchased from Boster (Wuhan, China). The kits to determine the activity of diamine oxidase (DAO) (Catalog number A088-2-1) and the concentration of D-lactic acid (D-LA) (Catalog number H263) in serum were purchased from Nanjing Jiancheng Bioengineering Institute (Nanjing, China). The kits to detect the concentration of TNF-*α* (Catalog number EK0527), IL-1*β* (Catalog number EK0394), IL-6 (Catalog number EK0411), and IL-10 (Catalog number EK0417) in serum were purchased from Boster (Wuhan, China). The anti-F4/80 antibody (Catalog number sc-377009) used for immunohistochemical analysis was purchased from Santa Cruz (Northern California, USA). The TUNEL kit (Catalog number 11684809910) used for in situ apoptosis degree determination was purchased from Roche (Basel, Switzerland). The anti-ZO-1 antibody (Catalog number ab221547) used for immunofluorescence analysis was purchased from Abcam (Cambridge, England). The protein extraction kit (Catalog number KGP2100) and butyl cyanoacrylate (BCA) kit (Catalog number KGP902) used for extracting and determining the concentration of protein were purchased from KeyGen (Nanjing, China). The primary antibodies used for determining the protein expression level of NF-*κ*B (p65) (Catalog number A00284-3), NF-*κ*B (p-p65) (Catalog number BM5404), Nrf2 (Catalog number PB9290), HO-1 (Catalog number BM4010), and GAPDH (Catalog number A00227) were purchased from Boster (Wuhan, China). The TRITC-conjugated goat anti-rabbit IgG for ZO-1 (Catalog number 111-026-047) and HRP-conjugated rabbit anti-goat IgG (Catalog number MBS584630) were purchased from JIR (Scottsdale, USA). The related primers used for real-time PCR analysis were synthesized by Sangon Biotech (Shanghai, China). The TRIzol reagent (Catalog number 15596018) used for RNA isolation was purchased from Invitrogen (Carlsbad, USA). The M-MuLV reverse transcriptase kit (Catalog number 18057018) was purchased from Fermentas (Glen BURNIE, USA). The SYBR Premix Ex Taq Kit (Catalog number RR420A) was purchased from Takara Biotechnology (Shiga, Japan).

### 2.2. Animal Experiments

**Figure 1:**
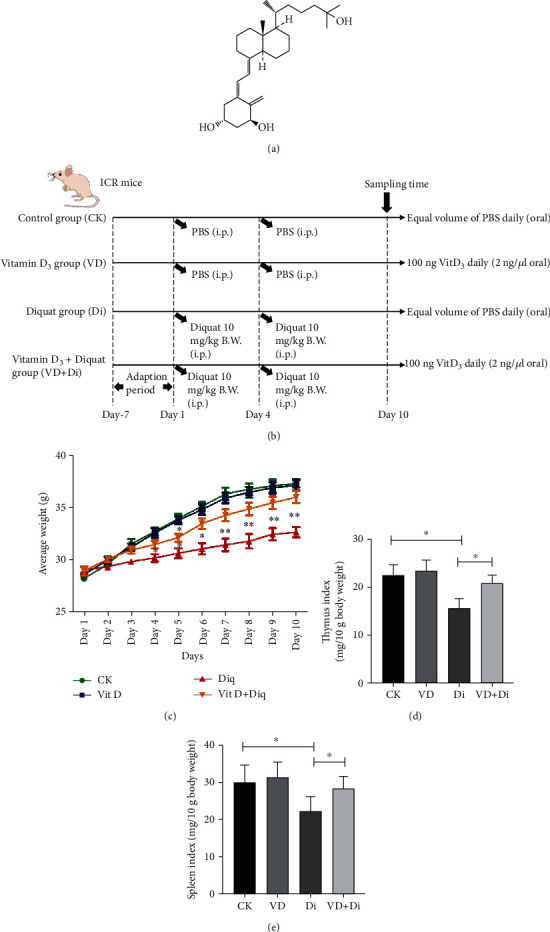
(a) The molecular structure of 1*α*,25-(OH)_2_-D_3_. (b) The experimental design and schematic diagram of treatments to each group. (c) Average body weight change of each group. (d) Thymus index and (e) spleen index of each group. All data were presented as mean ± SEM (*n* = 10). ^∗^*P* < 0.05, ^∗∗^*P* < 0.01.

The animal experiment was approved by the Animal Welfare and Ethics Committee of Hainan University (permit number: HNUAUCC-2020-00068) and conducted according to the National Institutes of Health guidelines for the care and treatment process of experimental animals strictly. Forty male ICR mice aged 7 weeks (24 ± 2 g) were purchased from the Experimental Animal Center of Hainan Medical School and raised for a one-week adaptation period. At the beginning of the formal experimental period, the mice were allotted to four groups (*n* = 10) randomly, as shown in the schematic diagram (Figure 1(b)): control (CK) group, VitD_3_ addition alone (VD) group, diquat-induced model (Di) group, and VitD_3_ addition+diquat (VD+Di) group. Firstly, the mice in the VD and VD+Di groups were administrated 100 ng VitD_3_ daily (1 mg VitD_3_ was dissolved in 1 mL 0.2% ethanol, and 2 *μ*L VitD_3_ solution was taken into 1 mL PBS to make the final concentration of VitD_3_ as 2 ng/*μ*L) through oral gavage. The other groups were given the same volume of PBS. On days 1 and 4, the Di and VD+Di groups were administered 10 mg diquat per kg B.W. suspended in 200 *μ*L volume of PBS through intraperitoneal injection. The other groups were injected with the same volume of PBS. Ad libitum feeding and drinking were assured during the experimental period. The average body weight gain of each group was recorded daily.

### 2.3. Sample Collection

On day 10, mice were sacrificed under the status of anesthesia through pentobarbital sodium injection. The blood was collected by breaking the posterior orbital venous plexus. The serum of each group was collected by centrifuging at 3500 rpm for 5 min under the temperature of 4°C. A part of the duodenum, jejunum, colon, liver, and kidney tissues was cut out and fixed with 4% paraformaldehyde immediately to prepare the paraffin section for further analysis. Another part of the jejunum was used as the tissue homogenate to determine the activity of antioxidase and the concentration of protein involved with antioxidant regulation. The residual jejunum tissue was frozen into liquid nitrogen immediately and then transferred to a -80°C refrigerator for further analysis of the relative expression level of the corresponding mRNA.

### 2.4. Determination of the Immune Organ Index

Before the execution of the mice, the individual weight of mice in each group was recorded, and the corresponding spleen and thymus were isolated and weighed without blood on organ surfaces (using absorbent paper to dry the surface blood). The immune organ index (IOX) was calculated using the following formula: IOX = weight of the immune organ (mg)/body weight (g) × 10.

### 2.5. Detection of the Oxidative Damage Index and Inflammatory Cytokines in Serum

The activities of ALT, AST, and DAO in serum were determined through an ELISA kit. The concentrations of D-LA, IL-1*β*, IL-6, TNF-*α*, and IL-10 in serum were determined using an ELISA kit. All the assays were carried out according to the manufacturer's instructions. To introduce briefly, the diluted samples were added to the appointed hole in the ELISA plate and mixed with the corresponding HRP-marked antibody. After the reaction lasted for 50 minutes, the reaction fluid in the ELISA plate was discarded and washed with PBS 5 times. Then, the substrate was added in order and reacted for 20 minutes under 37°C without light. Finally, the terminating fluid was added to stop the reaction and the OD value was recorded under the wavelength of 450 nm by a microplate reader (SpectraMax M5, Molecular Devices, USA).

### 2.6. Determination of Antioxidative Enzymes in the Serum and Jejunum

The activities of SOD, GSH-Px, and T-AOC in the serum and jejunum tissues were determined by an ELISA kit. The concentration of MDA in the serum and jejunum was analyzed by an ELISA kit. All the assays were carried out according to the manufacturer's instructions.

### 2.7. Evaluation of Morphology in the Duodenum, Jejunum, Colon, Liver, and Kidney

The middle site of the duodenum, jejunum, and colon and the representative part of the liver and kidney were separated to carry out hematoxylin and eosin (H&E) staining. Briefly, the samples taken out from 4% paraformaldehyde solution were embedded in paraffin and cut into slices. Then, the deparaffinized slices were stained with hematoxylin and eosin in turn. The morphological characteristics were observed using a Leica NEWDM 4500BR microscope (Leica, Frankfurt, Germany) under different magnifications.

### 2.8. Analysis of the Inflammatory Infiltration of Macrophages in the Jejunum Tissue

The middle part of the jejunum was isolated for immunohistochemical analysis of macrophage infiltration by a specific antigen marker of F4/80. The sections embedding in paraffin were deparaffinized and rehydrated with distilled water. 1% *w*/*v* BSA was applied to block the nonspecific binding sites for 30 min, and the anti-F4/80 antibody was incubated overnight at 4°C with the section at a dilution of 1 : 100. Further, the sections were washed with PBS and treated with HRP-conjugated rabbit anti-goat IgG at the ratio of 1 : 1000, incubated for another 1 h at 4°C, and washed with PBS.

### 2.9. Determination of the Apoptotic Level of the Jejunal Epithelium

The same part of the jejunum was used for the analysis of the apoptosis degree of the jejunal epithelium. To introduce briefly, the samples were deparaffinized and xylene was used to increase the transparency of slices. TdT and dUTP were mixed at a ratio of 1 : 9 and incubated with the slices for 60 min at 37°C. The endogenous peroxidase was blocked, and the slices were allowed to dry naturally. The slices were then covered with converter peroxidase and incubated for another 37°C for 30 min. DAB was added to the slices, and distilled water was used to stop color development. The cell nucleus was then stained using hematoxylin as a counterstain, and the slices were dehydrated and mounted.

### 2.10. Detection of the In Situ Expression Level of ZO-1 in the Jejunum Tissue

To determine the expression abundance of ZO-1 in the jejunum, the jejunum tissues kept in 4% paraformaldehyde were prepared as paraffin sections for immunofluorescence analysis. To introduce briefly, the slices were deparaffinized and antigen retrieval was carried out. Further, the slices were incubated with 3% hydrogen dioxide in the darkroom and then incubated with a primary antibody specific for ZO-1 at the dilution ratio of 1 : 200. Finally, TRITC-conjugated goat anti-rabbit IgG for ZO-1 was incubated with the slices for 1 h in the darkness and DAPI was then used to stain the nucleus directly. Images were taken under a Leica fluorescence microscope (Leica, Frankfurt, Germany).

### 2.11. Analysis of the mRNA Expression Level of Mucins and Tight Junction Proteins in the Jejunum

The residual jejunum tissues preserved in the -80°C refrigerator were taken out, and RNA samples were extracted using the TRIzol reagent. cDNA synthesis was conducted by the M-MuLV reverse transcriptase kit. The relative mRNA expression level of each gene was determined by the real-time PCR disposing process using the SYBR Premix Ex Taq Kit under the ABI StepOne Plus Real-Time PCR System (Applied Biosystems, CA, USA). Data was analyzed based on the comparative threshold cycle (Ct) method and normalized to the housekeeping gene GAPDH. The corresponding primers used in the experiment are listed in Table 1; the relative mRNA expression levels of mucins (Mucin-1, Mucin-2) and tight junction genes (ZO-1, ZO-2, Claudin-1, and Occludin) were determined on jejunum samples.

### 2.12. Detection of the Protein Expression Level of NF-*κ*B (p65)- and Nrf2-Related Proteins in the Jejunum

The protein extraction kit was used to isolate the total protein from the jejunum, and the concentration of protein was determined by the BCA assay kit. The protein expression levels of NF-*κ*B (p65), NF-*κ*B (p-p65), Nrf2, HO-1, and GAPDH were evaluated by western blot analysis according to the previous method reported [18]. The density of protein bands was visualized by the ECL substrate kit (Bio-Rad, CA, USA) through Champchemi 500 Plus (Saizhi, Beijing, China) and quantified using the ImageJ analyzer software.

### 2.13. Statistical Analysis

Multiple comparison tests were carried out by one-way analysis of variance with GraphPad prism (version 7.0, San Diego, USA). The value of *P* < 0.05 (^∗^) was considered significant, and the value of *P* < 0.01 (^∗∗^) was considered highly significant. Results were expressed as mean ± standard error (SEM).

## 3. Results

### 3.1. The Effects of VitD_3_ on the Growth Performance and Immune Organ Index Induced by Diquat

As shown in Figure 1(c), oral gavage of VitD_3_ alone did not affect the average weight during the experimental period. Compared with the CK group, the average weight of the Di group was significantly lower (*P* < 0.05) since day 4. From day 7 to day 10, the average weight between the Di group and the CK group showed a highly significant difference (*P* < 0.01). Meanwhile, the average weight of the VD+Di group showed a normal level except on day 5, which presented a significant difference (*P* < 0.05) compared with the CK group. As shown in Figures 1(d) and 1(e), the thymus index and spleen index of the Di group were significantly lower than that of both the CK and VD+Di groups (*P* < 0.05), which indicated the protective effect of VitD_3_ to immune organs.

### 3.2. The Effects of VitD_3_ on the Oxidative Damage Index Challenged by Diquat

**Figure 2:**
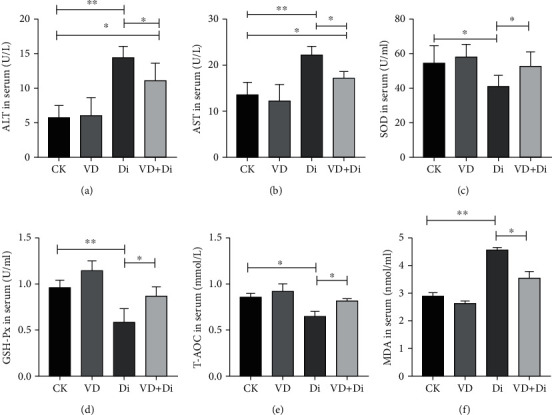
Effects of VitD_3_ on liver function and antioxidant capacity in mice. (a) ALT activity in serum. (b) AST activity in serum. (c) SOD activity in serum. (d) GSH-Px activity in serum. (e) T-AOC concentration in serum. (f) MDA concentration in serum. All data were presented as mean ± SEM (*n* = 10). ^∗^*P* < 0.05, ^∗∗^*P* < 0.01.

**Figure 3:**
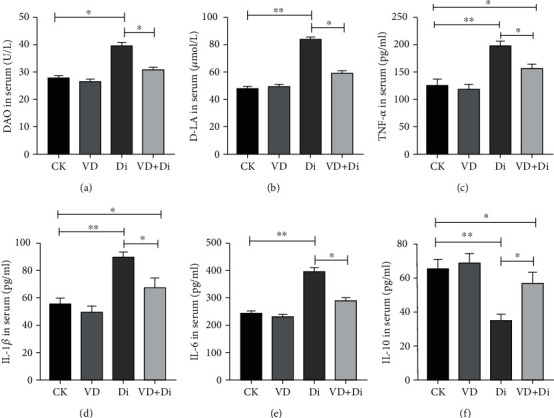
Effects of VitD_3_ on intestinal permeability and inflammatory cytokine secretion in serum of mice. (a) DAO activity in serum. (b) D-LA concentration in serum. (c) TNF-*α* concentration in serum. (d) IL-1*β* concentration in serum. (e) IL-6 concentration in serum. (f) IL-10 concentration in serum. All data were presented as mean ± SEM (*n* = 10). ^∗^*P* < 0.05, ^∗∗^*P* < 0.01.

As shown in Figures 2(a) and 2(b), the activities of ALT and AST in serum of the Di group showed a highly significant difference compared with those of the CK group (*P* < 0.01). Compared with the Di group, the activities of ALT and AST in serum of the VD+Di group were significantly lower (*P* < 0.05), and there remained a significant difference between the CK and VD+Di groups (*P* < 0.05). As shown in Figure 3(a), the activity of DAO in serum of the Di group was significantly higher than that of both the CK and VD+Di groups (*P* < 0.05). As to the concentration of D-LA in serum (Figure 3(b)), the Di group showed a highly significant difference compared with the CK group (*P* < 0.01), and the VD+Di group showed a significantly lower level than the Di group (*P* < 0.05). There remained a significant difference between the CK and VD+Di groups (*P* < 0.05).

### 3.3. The Effects of VitD_3_ on the Antioxidant-Related Index Challenged by Diquat

**Figure 4:**
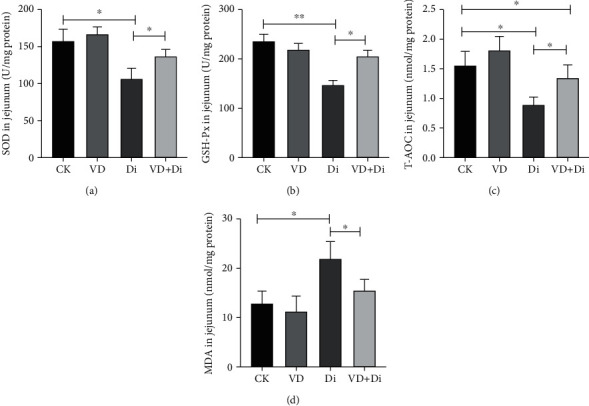
Effects of VitD_3_ on antioxidant capacity of the jejunum in mice. (a) SOD activity in the jejunum. (b) GSH-Px activity in the jejunum. (c) T-AOC concentration in the jejunum. (d) MDA concentration in the jejunum. All data were presented as mean ± SEM (*n* = 10). ^∗^*P* < 0.05, ^∗∗^*P* < 0.01.

As shown in Figures 2(c) and 4(a), the activities of SOD in the serum and jejunum of the Di group were significantly lower than those of the CK and VD+Di groups (*P* < 0.05). The activities of GSH-Px in the serum and jejunum of the Di group (Figures 2(d) and 4(b)) showed a highly significant difference compared with those of the CK group (*P* < 0.01), while the activities of the VD+Di group were significantly higher than those of the Di group (*P* < 0.05). The concentration of T-AOC in the serum and jejunum of the Di group (Figures 2(e) and 4(c)) was significantly lower than that of the CK and VD+Di groups (*P* < 0.05), while the concentration of T-AOC in the jejunum remained significantly different between the CK and VD+Di groups (*P* < 0.05). The concentration of MDA in serum of the Di group was significantly higher than that of the VD+Di group (*P* < 0.05), while it showed a highly significant difference compared with the CK group (*P* < 0.01) (Figure 2(f)). The concentration of MDA in the jejunum of the Di group was significantly higher than that of the CK and VD+Di groups (*P* < 0.05) (Figure 4(d)).

### 3.4. The Effects of VitD_3_ on the Secretion of Inflammatory Cytokines in Serum Induced by Diquat

As shown in Figures 3(c)–3(e), the concentrations of TNF-*α*, IL-1*β*, and IL-6 in serum of the Di group showed a highly significant difference compared with those of the CK group (*P* < 0.01) and were significantly higher than those of the VD+Di group (*P* < 0.05), while the concentration of TNF-*α* and IL-1*β* in serum of the VD+Di group was still significantly higher than that of the CK group (*P* < 0.05). As shown in Figure 3(f), the concentration of IL-10 in serum of the Di group showed a highly significant difference compared with that of the CK group (*P* < 0.01) and was significantly lower than that of the VD+Di group (*P* < 0.05), while the concentration of IL-10 in the VD+Di group remained significantly lower than that in the CK group (*P* < 0.05).

### 3.5. The Effects of VitD_3_ on the Intestinal Morphology Challenged with Diquat

**Figure 5:**
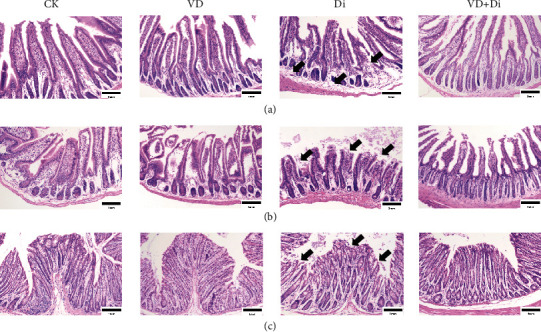
Effects of VitD_3_ on intestinal morphological characteristics. H&E staining of the (a) duodenum, (b) jejunum, and (c) colon tissues. The morphology of each segment was viewed at the magnification of 100x through a microscope.

As shown in Figures 5(a)–5(c), diquat caused severe injuries to the mucosal architecture of the duodenum, jejunum, and colon. As indicated by the black arrows, the Di group had sparse intestinal villi, shedding and truncated intestinal epithelia in the jejunum and colon, and pathological edema in the submucosa of the duodenum, while the VitD_3_-treated group prominently reversed the pathological characteristics described above.

### 3.6. The Effects of VitD_3_ on the Morphology of the Liver and Kidney Tissues Induced by Diquat

**Figure 6:**
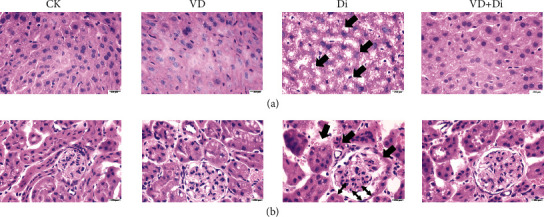
Effects of VitD_3_ on morphological characteristics of the liver and kidney. H&E staining of the (a) liver and (b) kidney. The morphology of each segment was viewed at the magnification of 100x through a microscope.

As shown in Figure 6(a), the hepatocytes were obviously vacuolated in the Di group, and the condensation of chromatin and nucleus swelling were also observed in the Di group (as indicated by the black arrows). Through the treatment of VitD_3_, the pathological features were largely improved. As shown in Figure 6(b), the Di group showed obvious degenerative changes in the tubules with exfoliated epithelia, pyknotic nuclei, and cystic dilatation (as indicated by the black arrows). The glomerulus was shrunken with wide Bowman's space (as indicated by the black double arrows), while minimal kidney tissue damage was observed in the VD+Di group.

### 3.7. The Effects of VitD_3_ on the Macrophage Infiltration and Epithelial Cell Apoptotic Level of the Jejunum Tissue Challenged with Diquat

**Figure 7:**
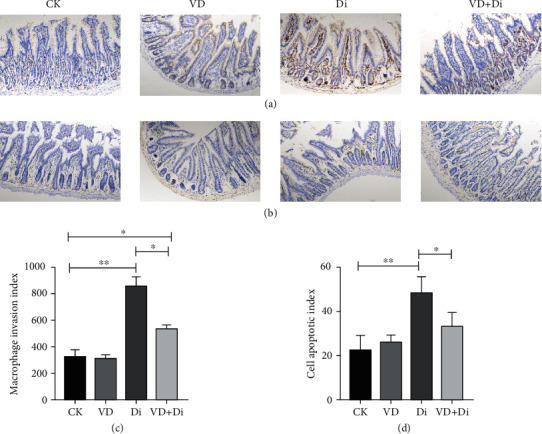
Effects of VitD_3_ on inflammatory infiltration of macrophages in the jejunum and cell apoptotic status in the jejunum. (a) Immunohistochemistry staining of macrophages (F4/80) in the jejunum tissue. (b) TUNEL staining of the jejunum tissue. All images were viewed at the magnification of 100x through a microscope. (c) Macrophage invasion index, which was calculated through collecting the integrated optical density (Image-Pro software) based on the positive reaction cells of at least six slices, and the average values were calculated and compared with each group. (d) Cell apoptotic index, which was calculated through collecting the integrated optical density (Image-Pro software) based on the positive reaction cells of at least six slices, and the average values were calculated and compared with each group. All data were presented as mean ± SEM (*n* = 6). ^∗^*P* < 0.05, ^∗∗^*P* < 0.01.

As shown in Figures 7(a) and 7(c), after the challenge of diquat, the infiltration of macrophages in the jejunum showed a highly significant difference between the CK and Di groups (*P* < 0.01), and the macrophage invasion index of the Di group was significantly higher than that of the VD+Di group (*P* < 0.05), while there remained a significant difference between the CK and VD+Di groups (*P* < 0.05). As shown in Figures 7(b) and 7(d), the cell apoptotic level of the jejunal epithelium in the Di group showed a highly significant difference compared with that in the CK group (*P* < 0.01), and the cell apoptotic index of the VD+Di group was significantly lower than that of the Di group (*P* < 0.05).

### 3.8. The Effects of VitD_3_ on the Expression Level of Intestinal Barrier Function-Related Genes in the Jejunum Induced by Diquat

**Figure 8:**
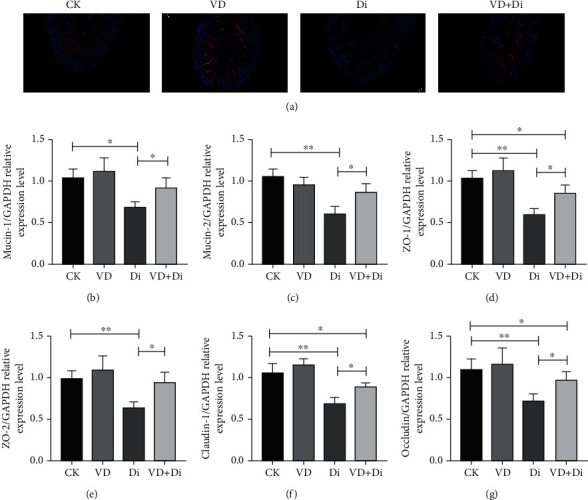
Effects of VitD_3_ on intestinal tight junction-related proteins and mucin expressions in the jejunum. (a) Immunofluorescence staining of ZO-1 protein in the jejunum tissue. The images were viewed at the magnification of 100x through a fluorescence microscope. Relative gene expression level of (b) Mucin-1, (c) Mucin-2, (d) ZO-1, (e) ZO-2, (f) Claudin-1, and (g) Occludin in the jejunum tissue. All data were presented as mean ± SEM (*n* = 6). ^∗^*P* < 0.05, ^∗∗^*P* < 0.01.

As shown in Figure 8(a), the expression abundance of ZO-1 protein in the Di group was visibly lower than that in the other three groups. The relative expression level of intestinal barrier function-related genes was determined (Figures 8(b)–8(g)); the relative expression levels of Mucin-2, ZO-1, ZO-2, Claudin-1, and Occludin in the Di group showed a highly significant difference compared with those in the CK group (*P* < 0.01), while the relative expression level of Mucin-1 in the Di group was significantly lower than that in the CK group (*P* < 0.05). The relative expression levels of related genes above in the VD+Di group were significantly higher than those in the Di group consistently (*P* < 0.05). The expression levels of ZO-1, Claudin-1, and Occludin in the VD+Di group remained significantly lower than those in the CK group (*P* < 0.05).

### 3.9. The Effects of VitD_3_ on the Inhibition of NF-*κ*B (p-p65) and Upregulation of Nrf2-Related Pathways Induced by Diquat

**Figure 9:**
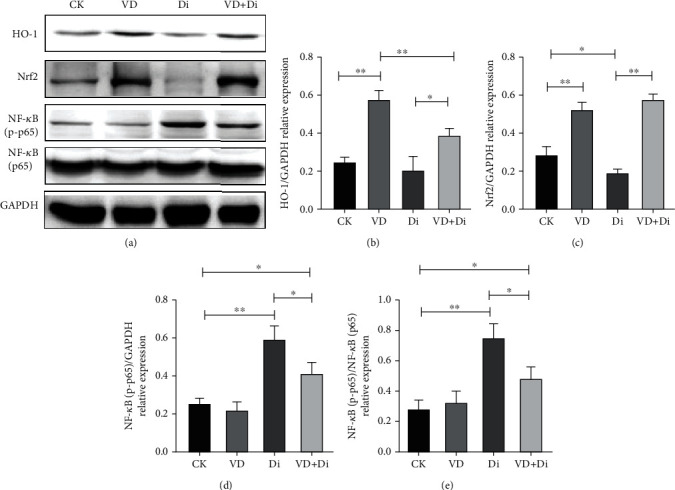
Effects of VitD_3_ on the Nrf2-mediated signaling pathway and phosphorylation level of NF-*κ*B (p65) in the jejunum. (a) The expression levels of HO-1, Nrf2, NF-*κ*B (p65), NF-*κ*B (p-p65), and GAPDH proteins detected by western blot analysis. Quantitative analysis of the protein expression levels of (b) HO-1, (c) Nrf2, and (d, e) NF-*κ*B (p-p65). All data were presented as mean ± SEM (*n* = 6). ^∗^*P* < 0.05, ^∗∗^*P* < 0.01.

As shown in Figures 9(a), 9(d), and 9(e), in the jejunum tissue, the phosphorylation level of NF-*κ*B (p65) showed a highly significant difference compared with the CK group (P<0.01), while the treatment of VitD_3_ significantly alleviated this trend (*P* < 0.05). As indicated in Figure 9(b), the addition of VitD_3_ could significantly promote the expression of HO-1 compared with the Di group (*P* < 0.05). From the result of Figure 9(c), the expression level of Nrf2 in the Di group was significantly inhibited compared with that in the CK group (*P* < 0.05), while the treatment of VitD_3_ enhanced the expression level of Nrf2 significantly (*P* < 0.05). The result also demonstrated that the treatment of VitD_3_ alone could significantly promote the expression level of Nrf2 and HO-1 compared with the CK group (*P* < 0.01).

## 4. Discussion

The main source of bioactive vitamin D (VD) to humans originated from plant-derived ergosterol (VD_2_) and animal-derived cholecalciferol (VD_3_) or exposure of the skin under the ultraviolet (UV) light [19]. Nowadays, there are at least 1 billion people that are diagnosed with deficiency of VD around the world in the 21st century, and the trend is increasing by year [20]. More studies support the point that VD not only keeps the health of the skeleton through promoting the absorption of calcium [21] but also has important functions except nutritional effects, including regulating the immune system to exert anti-infective effects and improving the developmental process of noninfectious diseases [22, 23]. The antioxidant function of vitamin C has been widely reported; however, the antioxidant effects and possible mechanism of VitD_3_have few studies.

In this study, the diquat-induced mouse oxidative stress model was established to explore the effects of VitD_3_ on the damage induced by diquat. In the study, we found that the challenge of diquat could reduce the average growth speed of mice, while the addition of VitD_3_ eliminated this symptom. The spleen index and thymus index are important indicators reflecting the status of innate immunity [24]; the lower immune organ index in the diquat group indicated the suppression of the normal development of the immune function, which may have a negative influence on the growth performance. The addition of VitD_3_ reverted the trend to the normal status, which refers to the positive effects of VitD_3_ on the immune function of the body. ALT and AST are important transaminases that are mainly synthesized in the liver, and the damage of hepatocytes will lead to the release of ALT and AST into the blood [25]. In this study, the abnormal rising of ALT and AST in serum of the Di group reflected the injury of diquat to the liver function, and it was inferred that the possible cause was the occurrence of oxidative stress. Diquat is a typical reagent which could lead to the overproduction of ROS in the body [14, 26] and is usually used to induce the oxidative damage model in animals [27, 28]. In this study, we consistently found that the activities of the main antioxidant enzymes in the serum and jejunum tissues of the Di group were significantly lower than those of the CK group, while the concentration of MDA, which is positively associated with the oxidative stress status [29], was significantly higher than that of the CK group. As the addition of VitD_3_ could efficiently ameliorate the antioxidant function in the serum and jejunum, we deduced that VitD_3_ may have the potential to regulate certain target proteins related to the redox balance.

Nrf2 is a crucial protein playing a key role in cellular defense against oxidative stress as the transcription factor [30]. It could effectively enhance the expression of antioxidant-related genes through binding to the antioxidant response elements (ARE) [31, 32]. The downstream functional proteins include SOD, GSH-Px, GST, and HO-1. In this study, compared with the CK group, we found that VitD_3_ could prominently enhance the protein expression abundance of Nrf2 and HO-1, no matter whether added alone or added with the challenge of diquat. Combined with the results determined on the level of antioxidant enzymes above, it can be inferred that VitD_3_ could activate the expression of Nrf2 and in turn promote the synthesis of antioxidant-related proteins. As a consequence, the damage caused by oxidative stress to the liver and kidney could be largely attenuated. There is increasing evidence supporting the hypothesis that the crosstalk occurs between the NF-*κ*B- and Nrf2-related pathways [33, 34]. NF-*κ*B (p65) could interact with Keap1 to inhibit the activation of the Nrf2-ARE pathway [35], which was confirmed once again in our present study. Meanwhile, the activation of the phosphorylation level of NF-*κ*B (p65) is positively associated with the secretion of proinflammatory cytokines such as IL-1*β*, IL-6, and TNF-*α* [36] and is negatively correlated with the secretion of anti-inflammatory cytokine IL-10 [37]. Our study further confirmed that the addition of VitD_3_ could distinctly lower the phosphorylation level of NF-*κ*B (p65) compared with the Di group. As a consequence, the addition of VitD_3_ effectively reverted the concentration of inflammatory cytokines in serum to be close to the normal state. The results above suggested that VitD_3_ could activate Nrf2-related pathways and inhibit the phosphorylation of NF-*κ*B (p65) to regulate the redox balance and inflammatory response simultaneously.

The occurrence of oxidative stress could directly damage the intestinal barrier function and cause dysfunction of digestion and absorption [38]. The abnormally high concentration of DAO and D-LA in serum always reflects the injury of intestinal barrier function and permeability [39]. In the study, the addition of VitD_3_ could effectively lower the concentration of DAO and D-LA in serum compared with the Di group, which suggested that VitD_3_ was involved in the improvement of intestinal barrier function. The description of the morphology of representative intestinal segments supported the point of view. As the jejunum is the main part which is responsible for the digestion and absorption of nutrients [40], keeping the healthy state of the intestinal barrier function of the jejunum tissue is crucial for normal growth performance. As tight junction proteins and mucins are the molecular basis of intestinal barrier structure [41], the expression level of the corresponding genes was determined. The results showed that diquat would destroy the structure of the intestinal barrier by inhibiting the expression level of the tight junction- and mucin-related genes, while the addition of VitD_3_ ameliorated the expression level of related genes above consistently. It is suggested that the amelioration of the imbalanced status of redox by VitD_3_ in the jejunum should be directly accounted for in this result.

The typical damage of oxidative stress to the intestine includes the invasion of inflammatory cells such as macrophages and neutrophils [42] and the increasing level of cell apoptosis in the intestinal epithelium [43]. In the study, we found that the diquat challenge caused serious infiltration of macrophages and the cell apoptosis degree in the intestinal epithelium of the Di group was visibly more severe than that of the CK group. The feeding of VitD_3_ reverted the pathological state to be close to the normal level. There may be two mechanisms that account for these results. One is the immunomodulatory function of VitD_3_ by inhibiting the phosphorylation level of NF-*κ*B (p65), which in turn lowered the local inflammatory level in the jejunum tissues. The other reason may owe to the antioxidant function of VitD_3_, which was realized by promoting the expression of Nrf2 and downstream proteins associated with enhancing the antioxidant capacity of the body. As a consequence, superfluous reactive oxygen species could be eliminated timely and the induction of cell apoptosis could be largely avoided. The liver and kidney are essential organs ensuring the synthesis of the active form of VitD_3_ [44], and the diquat challenge could cause serious damage to these two organs [13, 16, 45]. In this study, we decided to add the active form of VitD_3_ directly. The external addition of VitD_3_ could effectively replenish the deficiency of endogenous synthesis of VitD_3_ caused by the diquat challenge. The results suggested that the addition of VitD_3_ could improve the typical pathological morphology of the liver and kidney, which would in turn promote the recovery of the corresponding biological functions.

## 5. Conclusions

**Figure 10:**
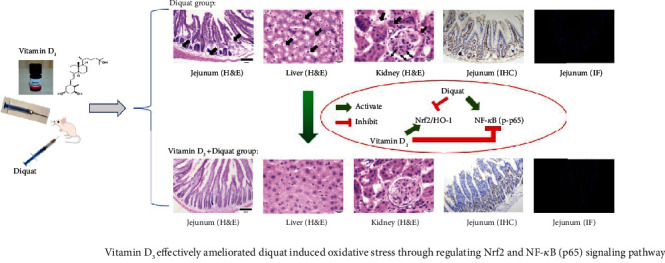
The conclusion of the effects of vitamin D_3_ on diquat-induced oxidative stress and the underlying mechanism.

In this study, we demonstrated that the exogenous supply of VitD_3_ could effectively alleviate diquat-induced oxidative stress and immune disorders. As a consequence, the intestinal barrier dysfunction and highly inflammatory status of the whole body were largely ameliorated. It was suggested that VitD_3_ may exert the function through activating the Nrf2-mediated signaling pathway while inhibiting the phosphorylation level of NF-*κ*B (p65) (Figure 10). Our findings offer important evidence to support that the additional supply of VitD_3_ in daily life could efficiently prevent oxidative stress-induced diseases, especially for those infants and older people who are susceptible to oxidative stress attacks.

## Figures and Tables

**Table 1:** The primers and sequences of qPCR.

Gene	Product size (bp)	Sequence (5′⟶3′)	Accession number (5′⟶3′)
GAPDH	212	F: GAGAAACCTGCCAAGTATGATGAC	NM_008084.3
ZO-1	198	R: TAGCCGTATTCATTGTCATACCAG F: TCATCCCAAATAAGAACAGAGC R: GAAGAACAACCCTTTCATAAGC	XM_006540786.1
ZO-2	269	F: GCTTTGGTGTGGACCAAGAT R: TCCATTATGGGTTTGCATGA	XM_006526909.1
Claudin-1	110	F: GCTGGGTCATCCTGGCTTCT R: CCTGAGCGGTCACGATGTTGTC	NM_016674.4
Occludin	86	F: CTTTGGCTACGGAGGTGGCTAT R: CTTTGGCTGCTCTTGGGTCTG	NM_006517566.1
Mucin-1	147	F: TGGATTGTTTCTGCAGATTTT R: CCTGACCTGAACTTGATGCT	NM_013605.2
Mucin-2	134	F: CCCAGAAGGGACTGTGTATG R: TGCAGACACACTGCTCACA	NM_023566.3

## Data Availability

The data used to support the findings of this study are available from the corresponding author upon reasonable request.
